# Signalling Three-Way Intersections: Is Redundancy Better Than Only Mandatory or Prohibitory Signs?

**DOI:** 10.3389/fpsyg.2021.712102

**Published:** 2021-10-26

**Authors:** Cristina Vargas, Sergio Moreno-Ríos

**Affiliations:** ^1^ERI-Lectura, University of Valencia, Valencia, Spain; ^2^The Mind, Brain and Behavior Research Center (CIMCYC-UGR), University of Granada, Granada, Spain

**Keywords:** mental models, three-way intersections, mandatory sign, prohibitory sign, redundant information

## Abstract

At intersections, drivers need to infer which ways are allowed by interpreting mandatory and/or prohibitory traffic signs. Time and accuracy in this decision-making process are crucial factors to avoid accidents. Previous studies show that integrating information from prohibitory signs is generally more difficult than from mandatory signs. In Study 1, we compare combined redundant signalling conditions to simple sign conditions at three-way intersections. In Study 2, we carried out a survey among professionals responsible for signposting to test whether common practices are consistent with experimental research. In Study 1, an experimental task was applied (*n*=24), and in Study 2, the survey response rate was 17%. These included the main cities in Spain such as Madrid and Barcelona. Study 1 showed that inferences with mandatory signs are faster than those with prohibitory signs, and redundant information is an improvement only on prohibitory signs. In Study 2, prohibitory signs were those most frequently chosen by professionals responsible for signposting. In conclusion, the most used signs, according to the laboratory study, were not the best ones for signposting because the faster responses were obtained for mandatory signs, and in second place for redundant signs.

## Introduction

When driving, we need to interpret mandatory and prohibitory traffic signs and make inferences to determine which direction is allowed and which is not. These inferences are made at the same time as many other cognitive activities we are engaged in. Therefore, it is not surprising that, at least under some circumstances, a large number of road accidents occur at intersections (see, for example, Pathivada and Perumal, 2019).

A potential way of reducing accidents at intersections is by applying the most suitable signposting policy to facilitate drivers’ inferences. For example, when we arrive at a T-junction where a right-turn is allowed, a valid traffic sign could be a mandatory sign for the right, a prohibitory sign for the left or both signs (a mandatory right-turn sign and a prohibitory left-turn sign). Although these three signing strategies may be equally valid from a legal point of view, the inferences required to decide which route is allowed involve a different burden on the cognitive system. Cognitive theories of thinking show that some inferences call for an intuitive system, aimed at making automatic fast inferences, while others require slow, effortful, more deliberative processing (e.g., Johnson-Laird, 1983; Stanovich, 1999; Evans, 2008; Kahneman, 2011; Khemlani et al., 2018).

In this work, we present further evidence regarding the use of mandatory and/or prohibitory traffic signs at intersections by considering the results of a new laboratory experiment on inference-making. In particular, we compared the effect of mixed redundant mandatory and prohibitory information to single-type conditions. In addition, we surveyed a group of professionals responsible for signposting policies to analyse consistency in the use of mandatory or prohibitory signs across different Spanish cities and also to examine whether their decisions were consistent with the results obtained in laboratory studies.

Previous literature analysed how people interpret mandatory and prohibitory traffic signs under different conditions by using a simple laboratory task (e.g., Castro et al., 2008; Vargas et al., 2011; Roca et al., 2012). In these experimental tasks, participants were generally presented with a traffic scene in which a car approaches a T-junction, with a road to the right and another to the left. A mandatory sign (e.g., right-turn) or a prohibitory sign (e.g., no left-turn) was shown, allowing only one direction. Subsequently, a car was shown in a new scene on one of the two possible roads (e.g., on the road to the right). Participants had to decide as quickly as possible whether the manoeuvre taken was allowed or not-allowed.

**Figure 1:**
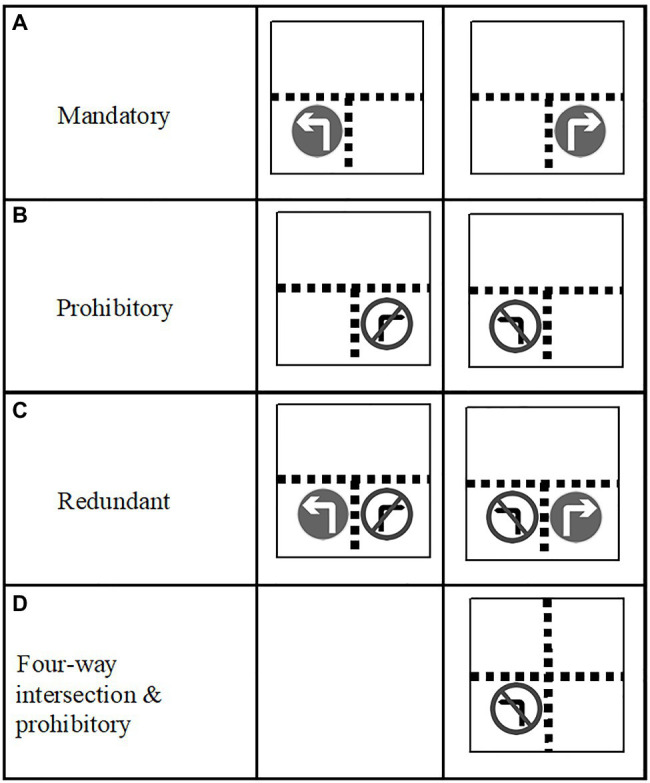
This graph displays types of sign and intersection. **(A)** Two examples of mandatory signs at a T-junction. **(B)** Two examples of prohibitory signs at a T-junction. **(C)** Two examples of redundant information (mandatory sign and prohibitory signs) at a T-junction. **(D)** One example of prohibitory sign at a four-way intersection.

This task has been successfully used to analyse how people make inferences, based, in particular, on predictions from the mental model theory (or model theory; Johnson-Laird and Byrne, 2002; see Johnson-Laird and Ragni, 2019). The model theory maintains that propositional and visual premises are converted into iconic representations called mental models. At a T-junction, a mandatory right-turn sign and a prohibitory left-turn sign may be equivalent in that both allow a right-turn (see Figures 1A,B), but their initial mental representations (initial models) are different. In the first case (mandatory right), the initial model represented would be

1. “Right”

while in the second case (prohibitory left), the initial model represented would be

1. “[prohibited] Left.”

However, in both cases, an explicit model (i.e., a full representation of each piece of information, including additional information) can be inferred and will be the same for both signs:

1. “Right allowed and left not-allowed.”

Thinking with the initial representations is faster and less error prone than thinking with explicit models, which requires effort (see Khemlani et al., 2018). Therefore, predictions in the experimental task described above were clear: participants would be faster in deciding that a road was allowed when it was signalled by a mandatory right-turn sign and also faster in deciding that it was not-allowed from a prohibitory left-turn sign. Results in different experiments confirmed such predictions. That is, the mandatory sign led to faster responses to the allowed road than the not-allowed one, and the prohibitory sign led to faster responses to the not-allowed road than the allowed one (Castro et al., 2008; Vargas et al., 2011; Roca et al., 2012).

Moreover, different factors that could modulate inferences with these signs have been studied, such as the number of roads at the intersection and the number of traffic signs (Castro et al., 2008), or the exposition time of the signs (Vargas et al., 2011).

Firstly, the advantage of the signalled road persisted at an intersection with four ways (see Figure 1D) instead of three (Castro et al., 2008). Also, the presentation of two signs (for example, a double prohibition: for the road ahead and to the right) as two isolated signs or two signs embedded in one did not show any difference in time of response or pattern of results. More interestingly, when people had to collect information from two mandatory signs (in both conditions, two isolated signs and two signs embedded in one), it led to faster responses than from two prohibitory signs. Therefore, for both regulatory signs, it was easier to respond that a turn was allowed than it was to give a not-allowed response (see Castro et al., 2008; Experiment 2). Besides, the disadvantage of using prohibitory signs cannot be explained just in terms of interference between the perceptual arrow direction and readiness to respond to the location tested. Roca et al. (2012; Experiment 2) replicated previous results controlling the Simon effect. Moreover, Castro et al. (2008) used directional and non-directional prohibitory signs. The magnitude of the effect did not differ between them. From a theoretical point of view, the prohibition on acting implies not acting (Johnson-Laird and Ragni, 2019). Some authors have proposed that prohibition requires adding a “mental footnote” (between brackets: “[Prohibited] left”) that is not present in the mandatory information (see Bucciarelli and Johnson-Laird, 2005; Vargas et al., 2011). This makes it more difficult to integrate information from premises with prohibition than with mandatory information.

Secondly, in Vargas et al. (2011), the time factor was manipulated in three experiments where encoding time (the time that the first scene was displayed) and signs exposure time (different display times for traffic signs were presented) were assessed in the first scene. The second scene, with the car in the target position, was always shown after the first scene had disappeared. Thus, participants had to make the decision as soon as the first scene disappeared. Participants showed that sign exposure time was irrelevant for making the inference: what was relevant was the total time given for processing the information (encoding time) in the first scene before having to give a response in the second scene. In this case, important differences were obtained according to whether participants had 300ms to comprehend the sign (encoding time) or 1,000 or 2,000ms. In all cases, the mandatory sign led to faster responses to the allowed road than the not-allowed one. Prohibitory signs led to the opposite, replicating the previous experiment in the short time condition. However, when participants had enough time (1,000 and 2,000ms conditions), they showed faster responses to the allowed road than the not-allowed road, as happened with the mandatory signs, but more slowly in both conditions (allowed and not-allowed; Vargas et al., 2011). Results are consistent with the proposal that the negation implied by the prohibition requires (as would “falsity”) a conversion of the information for taking one road to the possibility of going the other way, which takes extra time and is error prone (see Bucciarelli and Johnson-Laird, 2005; Vargas et al., 2011; Espino and Byrne, 2018; Moreno-Ríos and Byrne, 2018).

To summarise, the results of the previous laboratory studies show that in simple situations, mandatory signs are better for signing allowed roads than not-allowed roads and the opposite happens for prohibitory signs. However, in general, it is difficult to maintain such an advantage of the prohibitory sign because, if participants have enough time, they will convert the prohibitory information into mandatory information. In complex situations, the integration of prohibitory information is more difficult and takes longer due to the complexity of the tasks required.

In one of the previous studies described above (Castro et al., 2008), the combination of two traffic signs was examined, but the effect of including mixed redundant information was not tested. For example, in our initial example at a T-junction (see Figure 1C), both mandatory right-turn and prohibitory left-turn signs could be used to reinforce the same message: only a right-turn is allowed. Obviously, by presenting two traffic signs, the amount of information available is greater, thus increasing the processing requirements and potentially making the task more difficult. Also, participants might check only one of the two signs and the global result could be a combination of responses to the single signs. Consequently, it is uncertain whether the effect of mixed redundancy would be positive or negative in this particular situation. In contrast, T-junctions provide the simplest condition to test the redundancy effect. By using T-junctions rather than including other junctions, some factors can be more easily controlled, such as the same number of different signs (one mandatory and one prohibitory) and ensuring that all the ways are signalled (avoiding the need to infer any other way). In addition, the two signs can be shown in just one location, which is not possible with four-way junctions.

Regarding the overall traffic literature, some previous studies on redundancy have shown that when signs are unfamiliar, the inclusion of a redundant text could improve comprehension of the signs and reduce the time for interpretation (Shinar and Vogelzang, 2013). In addition, there are some mixed results regarding the effect of redundancy in Variable Message Signs, in particular, about whether it reduces compliance with the target detour message (Thomas and Charlton, 2020) or not (Harms et al., 2019).

Looking at the current traffic literature, we considered that no accurate predictions could be made at this point regarding the use of redundancy when presenting mandatory or prohibitory traffic signs at T-junctions. Therefore, we first carried out an experimental study (Study 1), in which we used a task similar to the one applied in previous studies, but now aimed at testing the potential usefulness of a mixed redundant double sign condition (Objective 1).

Second, trying to expand the results found in the laboratory to real situations, we carried out a survey (Study 2) to evaluate current policies on signposting in different cities across Spain. In particular, we tested whether there is agreement among professionals responsible for signposting when they are designing three-way and four-way intersections (Objective 2) and whether those practices are consistent with the reported experimental results (Objective 3).

## Study 1

Previous studies have shown that the integration of two mandatory signs was easier than the integration of two prohibitory signs (Castro et al., 2008). However, no previous study has been done with redundant information (i.e., a mandatory and a prohibitory sign). From the mental model theory, the two signs lead participants to two initial representations that cannot be directly integrated because they do not share the initial representation, as shown in models (1) and (2) (see section “Introduction”). Only with the complete representation of the explicit model (3) can they be integrated, providing confirmation that both signify the same. Therefore, model theory is useful for making predictions regarding the use of redundancy when combining mandatory and prohibitory traffic signs. From this theoretical point of view, an overall delay is expected regarding the initial representation obtained with the mandatory or prohibitory signs (specifically, allowed for mandatory signs and not-allowed for prohibitory signs conditions) because the redundant condition requires the construction of two models. In addition, when the response entails accessing the explicit model (that is, inferring the not-allowed road for mandatory signs and the allowed road for prohibitory signs conditions), faster responses would be expected in the redundant condition. These results are expected when participants process both signs, but it is important to note that with redundant information, they could also attend to just one of the two signs systematically (e.g., the mandatory sign). In this case, no differences would be expected between the redundant condition and the simple condition (mandatory or prohibitory sign).

Thus, in this study, we examine the effect of redundancy by comparing three equivalent traffic scenes (mandatory, prohibitory, and redundant signs) at a T-junction. In addition, we will analyse the effect of exposition time (300 vs. 1,000ms) to test the robustness of the effect found by Vargas et al. (2011).

### Materials and Methods

#### Participants

Twenty-four students (21 females) participated in the experiment. They were either Psychology or Speech and Language Therapy students at the University of Granada (Spain). Average age was 21.2 (*SD*=4.2). They all had normal or corrected-to-normal vision and received course credits as compensation for their participation.

#### Stimuli

**Figure 2:**
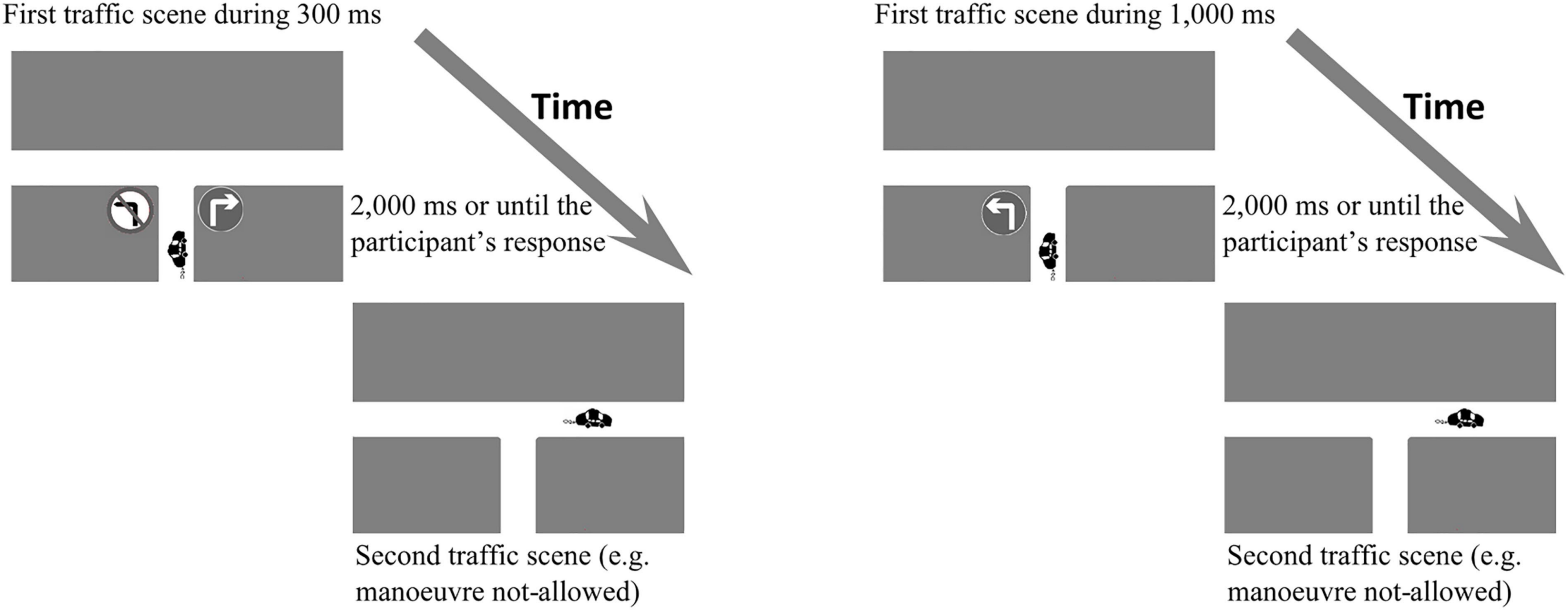
Examples of the consecutive traffic scenes at a T-junction shown in Study 1.

The procedure used in Study 1 was similar to Experiment 1 in Vargas et al. (2011). T-junction road traffic scenes were used in this experiment. Two consecutive screens were shown in each trial. The first scene was presented with a mandatory, a prohibitory or a redundant (one mandatory and one prohibitory) traffic sign for 300 or 1,000ms. After that period, a second scene was displayed for a maximum of 2,000ms or until the participant responded (Figure 2). In the first scene, two-thirds of the cases presented a single traffic sign (either mandatory or prohibitory) and the remaining third presented both types of sign (redundant condition). Figures 1A–C show the different combinations of signs in this experiment. An E-Prime software (Schneider, 2003) script was developed to control the representation of stimuli and the collection of responses on a 15-in. PC screen.

#### Procedure

First, the participants read and signed an informed consent form. After that, participants carried out the experiment individually, seated in front of a computer screen. Instructions for the experiment were shown. These instructions explained that the experiment consisted of evaluating the events shown in two consecutive traffic scenes. The participants were informed that the first scene always showed a car on the lower street with various roads it could take and one or two traffic signs. The second scene showed the same car arriving at one of the two other roads at the T-junction (left or right). After that, participants were asked to evaluate whether the road taken by the car was allowed or not-allowed according to information provided by the sign(s).

Participants had to press the “Z” key, labelled as “allowed”, as quickly as possible if the manoeuvre was allowed. If the manoeuvre made by the car was not-allowed according to the sign(s), the “M” key, labelled as “not-allowed”, had to be pressed. The response key was counterbalanced across participants. Feedback was provided about whether the correct or incorrect response had been performed.

There were 12 experimental conditions defined by combining the time of exposure for traffic signs (300 and 1,000ms), type of sign (mandatory, prohibitory and redundant) and manoeuvre (not-allowed and allowed) as variables. After reading the instructions, participants performed a block of 48 practice trials (four trials per experimental condition) followed by four blocks of 72 experimental trials (six trials per experimental condition). Thus, the total number of experimental trials was 288 (24 per experimental condition). The order of stimuli presentation was determined randomly for each block.

### Results and Discussion

Trials with RTs above and below three SDs across each participant and condition were excluded from the analysis. This removes the outliers that occur when participants do not follow the instructions or do not perform the task. This criterion resulted in 0.86% of the trials being eliminated from the analysis. A repeated measures ANOVA was conducted to analyse mean RTs for correct responses. The ANOVA included time of exposure to traffic signs (300 vs. 1,000ms), type of sign (mandatory vs. prohibitory vs. redundant) and manoeuvre (not-allowed vs. allowed). Prior to the ANOVA, data were tested with Mauchly’s test of sphericity and degrees of freedom modified as necessary. We used the Bonferroni test to carry out planned comparisons. All analyses were performed using the IBM SPSS Statistics 24 software.

Mean correct response times (RTs) and SDs for each main effect are shown in Table 1.

**Table 1:** Mean correct RTs (ms) and SDs (in parentheses) for each main effect in Study 1.

	Type of sign			Manoeuvre		Overall average
	Mandatory	Prohibitory	Redundant	Not-allowed	Allowed	
300ms	604.9 (181.9)	702.3 (185.3)	642.6 (187.5)	692.7 (189.2)	607.2 (180.1)	649.9 (182.3)
1,000ms	505.2 (166.7)	609.6 (175.6)	543.9 (178.4)	587.6 (178.6)	518.2 (163.5)	552.9 (170.0)
Overall average	555.1 (171.8)	655.9 (177.8)	593.2 (179.9)	640.2 (181.9)	562.7 (170.1)	601.4 (174.7)

**Figure 3:**
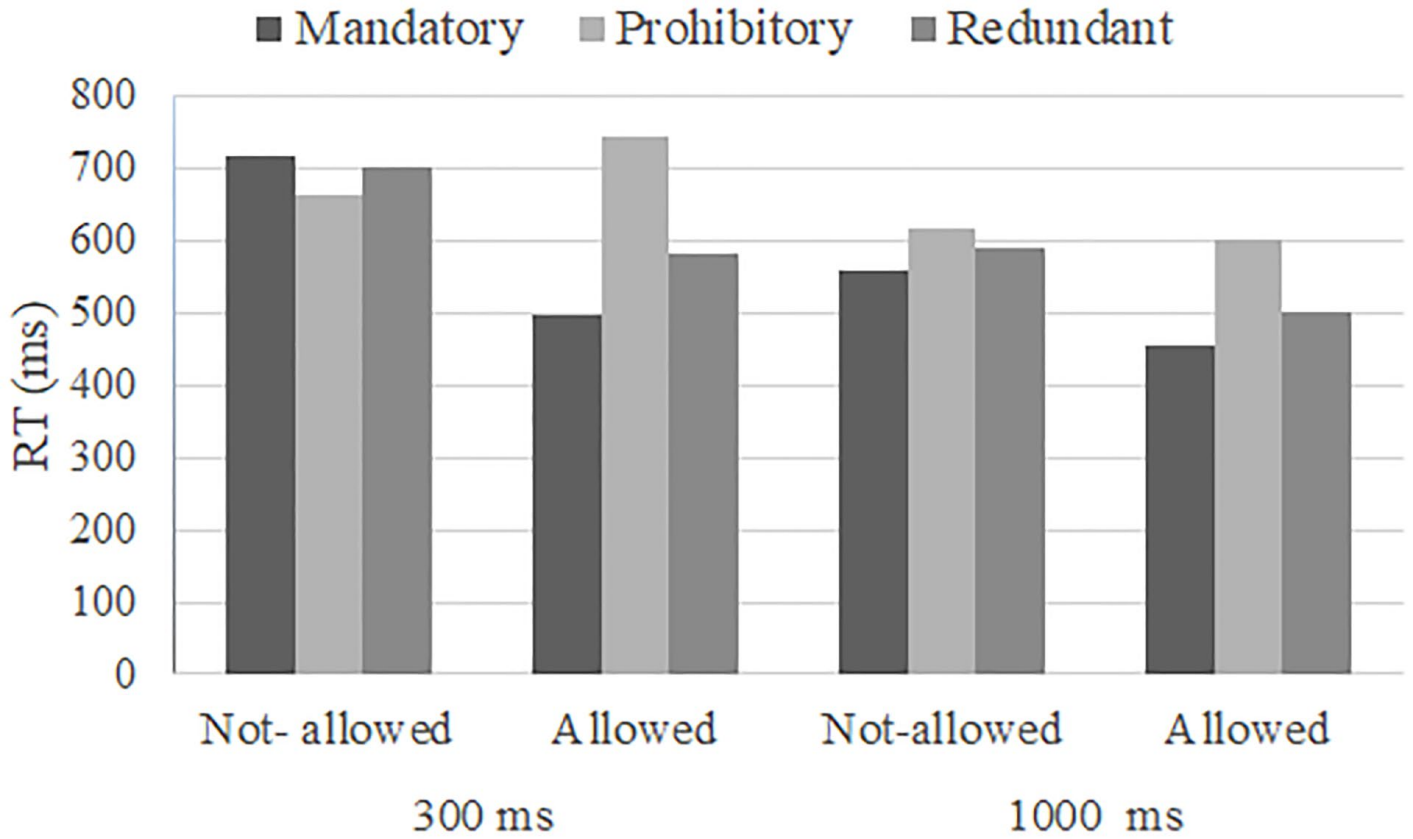
Mean reaction time for the conditions manipulated in Study 1: time of exposure to traffic signs (300 vs. 1,000ms), type of sign (mandatory vs. prohibitory vs. redundant) and manoeuvres (not-allowed vs. allowed).

As expected, the second-order interaction (exposure to traffic signs×type of sign×manoeuvre) was statistically significant, *F*(2,46)=25.953, *p*<0.001, *η*^2^=0.53 (Figure 3 shows graphically the second-order interaction). In this context, time of exposure to traffic signs modulates the interaction between type of sign and manoeuvre. These differences are much stronger for the shorter duration (300ms condition). In order to analyse this second-order interaction, further separate analyses were carried out for the 300 and 1,000ms conditions, in accordance with Vargas et al. (2011).

The analysis of the 300ms experimental condition revealed a significant interaction between the type of sign and manoeuvre, *F*(1.533, 35.251)=54.913, *p*<0.001, *η*^2^=0.71. We carried out planned comparisons of type of sign and manoeuvre interaction. First, we reproduced previous results by simple contrast (not-allowed vs. allowed for each type of sign: mandatory, prohibitory and redundant). There were statistically significant differences for mandatory signs (*Bonferroni*=219.314, *p*<0.001), prohibitory signs (*Bonferroni*=−82.520, *p*<0.001) and redundant signs (*Bonferroni*=119.902, *p*<0.001). On average, participants were faster when an allowed manoeuvre was presented, as compared to a not-allowed manoeuvre, but only for mandatory and redundant signs conditions. In other words, redundant signs have the same effect as mandatory signs. The opposite was true for prohibitory signs (see Figure 3). Thus, previous findings were replicated. Second, in order to test Objective 1 in the case study, a simple contrast for manoeuvre (mandatory vs. prohibitory vs. redundant for each manoeuvre) was performed. Results showed statistically significant differences for the allowed condition, but not for the not-allowed condition. The analysis of the allowed condition revealed statistically significant differences between the following experimental conditions: mandatory vs. redundant signs (*Bonferroni*=−87.447, *p*<0.001), redundant vs. prohibitory signs (*Bonferroni*=−160.871, *p*<0.001) and mandatory vs. prohibitory signs (*Bonferroni*=−248.318, *p*<0.001). Consequently, when an allowed manoeuvre was evaluated, participants’ responses were faster for mandatory signs, followed by redundant signs, and finally, prohibitory signs. Moreover, the analysis of the 300ms experimental condition revealed main effects of type of sign [*F*(2, 46)=35.918, *p*<0.001, *η*^2^=0.61], and of manoeuvre, [*F*(1, 23)=49.201, *p*<0.001, *η*^2^=0.68; see Table 1].

Regarding the 1,000ms experimental condition, the type of sign and manoeuvre interaction was also significant, *F*(2, 46)=9.108, *p*<0.001, *η*^2^=0.28. To examine this interaction further, in a similar way to the analysis of the 300ms condition, we first carried out a simple contrast test for type of sign (not-allowed vs. allowed for each type of sign). There were statistically significant results for mandatory signs (*Bonferroni*=102.725, *p*<0.001) and redundant signs (*Bonferroni*=88.854, *p*<0.001), but not for prohibitory signs. On average, participants responded faster to allowed manoeuvres for mandatory and redundant signs conditions than to not-allowed manoeuvres. However, there were no differences between manoeuvre types (not-allowed vs. allowed) for prohibitory signs (see Figure 1). Thus, the results of the aforementioned studies were replicated once again. Second, a simple contrast for manoeuvre (mandatory vs. prohibitory vs. redundant for each manoeuvre) was performed. Results showed statistically significant differences for both conditions of manoeuvre. For the not-allowed condition, there was a statistically significant difference between mandatory and prohibitory signs (*Bonferroni*=−61.346, *p*<0.01). That is, participants responded faster to mandatory signs than to prohibitory signs for a not-allowed manoeuvre. Regarding the allowed condition, statistically significant results were found for all experimental comparisons: mandatory vs. redundant signs (*Bonferroni*=−45.583, *p*<0.01), redundant vs. prohibitory signs (*Bonferroni*=−101.841, *p*<0.001) and mandatory vs. redundant signs (*Bonferroni*=−147.424, *p*<0.001). As in the 300ms condition, participants showed the fastest performance on average when a mandatory sign was presented, followed by redundant signs, and finally, prohibitory signs. Regarding main effects of both type of sign and manoeuvre in the 1,000ms condition, the analysis revealed significant main effects of the type of sign [*F*(2, 46)=34.317, *p*<0.001, *η*^2^=0.60] and of manoeuvre, [*F*(1, 23)=67.797, *p*<0.001, *η*^2^=0.75; see Table 1].

Finally, the overall main effects of all three independent variables were statistically significant: time of exposure to traffic signs [*F*(1, 23)=105.133, *p*<0.001, *η*^2^=0.82], type of sign, [*F*(2, 46)=62.987, *p*<0.001, *η*^2^=0.73] and manoeuvre [*F*(1, 23)=75.390, *p*<0.001, *η*^2^=0.77]. Hence, on average, participants responded faster to the 1,000ms than 300ms condition, mandatory vs. redundant signs, redundant vs. prohibitory signs and allowed vs. not-allowed (Table 1).

There was no trade-off effect, that is, no correlation was found between reaction times and accuracy scores. We found accuracy measures with more than 86.9% of answers correct. The low frequency of errors led to a limited window for effects. Therefore, the accuracy measures are not shown in the manuscript.

According to the results of Study 1, the strategy used to process the combined condition differed depending on the manoeuvre being evaluated. For the not-allowed condition, there were no statistically significant differences between redundant and prohibitory signs (initial model) nor between redundant and mandatory signs (explicit model), which suggests that the preference was to specifically focus on one of the two signs of the combined condition.

In contrast, when the manoeuvre being evaluated was allowed, we observed statistically significant differences between the single and the combined signs conditions, which suggests that participants were actually processing both signs. This result is consistent with the predictions of mental model theory in both the 300 and 1,000ms times of exposure to traffic signs: (a) when the initial model was represented, participants were faster for mandatory than for redundant signs; and (b) the pattern was reversed for the explicit model, that is redundant signs achieved faster responses than prohibitory signs.

In addition, the present outcome replicates previous results regarding the time for processing conditions. As in Vargas et al. (2011), when participants had little time to process the premises, the initial representation of the mandatory sign led participants to react faster for the allowed way than for the not-allowed one, and the opposite result was obtained with prohibitory signs. Results showed that when the two signs were used in this condition, results were similar to those for the mandatory sign (with slightly slower times in all average conditions). Also, as in previous studies, when participants were given longer to process the information (1,000ms), the advantage for faster responses for the allowed road than the not-allowed remained for mandatory signs, and this also happened with the two redundant signs, but the difference between the two conditions disappeared for the prohibitory signs, which took longer than with the other two kinds of signing.

The most striking feature of these results is that, in the tested conditions, significantly faster responses were generally obtained with the mandatory signs, while there was no advantage for using mixed redundant or prohibitory signs. Regarding prohibitory signs, the performance was not significantly better than when the other two kinds of signing were used, while the redundant signs showed better results than prohibitory ones only in some particular experimental conditions (i.e., allowed condition).

## Study 2

As suggested by the previous research, there is generally an advantage in using mandatory signs at intersections. Therefore, in Study 2, we surveyed professionals responsible for signposting in provincial capitals of Spain to analyse traffic signs used at intersections and to identify some of the factors modulating their decision-making (e.g., a recent change of direction in the road, accidents reported…). In particular, we tested whether the different professionals responsible for signing: (a) make similar decisions when signalling three and four-way intersections and (b) whether their decisions are consistent with the current experimental research.

### Materials and Methods

#### Participants

We conducted a cross-sectional survey. The population of interest were professionals responsible for signposting in Spanish provincial capitals. An email was sent to administration staff, inviting them to take part in the study, entitled “Use of obligation and prohibition traffic signs”. In a 10-week period, nine survey respondents completed all the questionnaires. Hence, the response rate was 17% (response rates to email-only surveys are seldom more than 20%, according to Dillman et al., 2014). These included among others (see section “Results and Discussion”), the most populated cities in Spain (such as Madrid, Barcelona and Valencia, with 14.9 million inhabitants in the three cities and their metropolitan areas in 2020; European Statistical Office, 2021). The average age of participants was 51.8years (*SD*=7.5), and they were all men. In addition, the majority of them were engineers.

#### Procedure and Survey

The survey was an online version, self-completed using LimeSurvey. The first version of the questionnaire was piloted with two road signing professionals (officials from Valencia City Council’s Department of Traffic) and an expert in traffic research (University of Valencia). We incorporated all their suggestions. In the final questionnaire, participants provided the following information: sex, age, city, traffic regulations applied (e.g., the General Traffic Regulations), which traffic sign was used more (prohibitory or mandatory) in general, and at three- and four-way intersections, and how often they used mandatory signs in the latter circumstance. In addition, participants were asked to indicate the most frequent signs utilised to obligate drivers in five different traffic situations (e.g., road with high density of traffic). The respondents had several options for response: “prohibitory”, “mandatory”, “prohibitory and mandatory”, “neither sign”, “other (to be specified)”. All survey respondents answered the same questions in the same order.

### Results and Discussion

**Figure 4:**
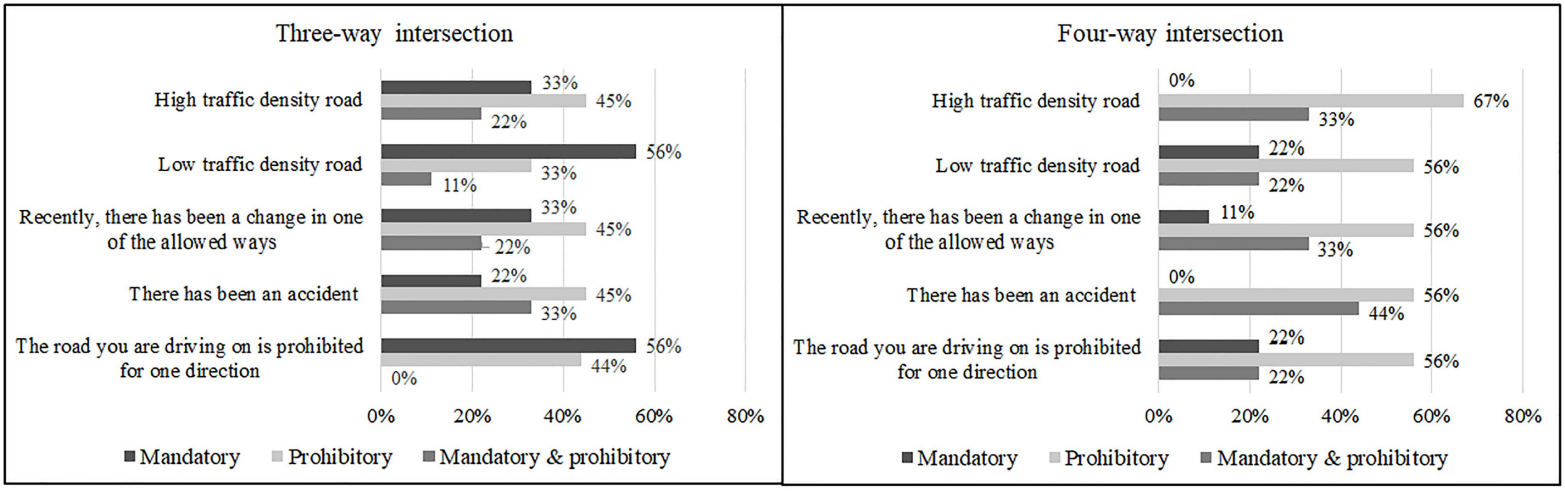
Percentage of choices of signs used most frequently to signpost at three and four-way intersections in different conditions.

The following provincial capitals took part in the research: Barcelona, Cádiz, Castelló de la Plana, Córdoba, Logroño, Madrid, Málaga, Valencia and Vitoria-Gasteiz. Eight out of nine respondents used the General Traffic Regulations (Reglamento General de Circulación) for applying mandatory and prohibitory traffic signs. None of the participants chose the categories of response “neither sign” or “other (to be specified)”, and therefore, only “prohibitory”, “mandatory,” and “prohibitory and mandatory” were considered. In general, the prohibitory signs were used slightly more than mandatory signs (55.6 and 44.4%, respectively). Among those who used the most prohibitory signs, the mandatory signs were utilised with a mean value of 25%. This percentage changed according to whether they applied these traffic signs to three-way or four-way intersections. In the first situation, 33.3% used prohibitory signs more frequently than mandatory signs. However, that value increased to 77.8% for four-way intersections. Finally, survey respondents applied different criteria according to the traffic conditions. For example, when there were more complicated traffic situations, the percentage of prohibitory signs used rose (Figure 4).

We were also interested in testing whether participants changed their choice of signalling from three-way intersections to four-way, and therefore, we evaluated the consistency of their responses across three-way and four-way intersections (Table 2). An exact multinomial test for paired contingency tables was applied and the results showed that it was not symmetrical (*p*<0.001). In addition, we carried out pairwise comparisons with multiple testing adjustment. According to these analyses, there was a significant change from mandatory to prohibitory signs (*p*<0.001; Cohen’s *g*=0.5) and from prohibitory to redundant signs (*p*=0.031, Cohen’s *g*=0.5). The analyses were carried out using the EMT and rcompanion packages implemented in R 3.6.3 (R Core Team, 2020).

**Table 2:** Cross-tabulation table of traffic signs changed from three-way intersections to four-way intersections.

Three-way intersection	Four-way intersection		
	Mandatory	Prohibitory	Mandatory and prohibitory
Mandatory	5	13	0
Prohibitory	0	13	6
Mandatory and prohibitory	0	0	8

Participants responsible for signposting did not follow the same strategies when deciding whether mandatory or prohibitory signs should be used in different conditions. Some of them systematically preferred to use prohibitory signs and others mandatory ones, but an overall preference for using prohibitory signs was found.

This result contrasts with those previously reviewed, obtained in the laboratory, which showed faster processing when inferences were based on mandatory signs than on prohibitory ones (Castro et al., 2008; Vargas et al., 2011; Roca et al., 2012; and Study 1).

In the case of four-way intersections, participants changed their criteria from those used for three-way intersections, increasing the number of prohibitory signs and decreasing the number of mandatory signs (from 18 mandatory signs at three-way intersections, 13 were changed to prohibitory at four-way intersections). In all cases, more prohibitory signs were used. This made it more probable that drivers would need to integrate the prohibitory information, yet mandatory signs are the most informative, given that they eliminate a greater number of alternatives.

Participants decided to use mandatory signs more frequently than prohibitory signs in only two conditions and only at three-way intersections: roads with low traffic density and roads where there was a not-allowed turn (i.e., when the allowed way was the more probable objective of drivers).

Another interesting result is that they decided to use the two signs more often at four-way intersections than at three-way ones. As we have noted in Study 1, in a particular case, using redundant signs showed better results than using prohibitory signs (i.e., allowed condition). The use of these signs happens more frequently when an accident has occurred there. In those situations, it is possible that visual material effects of the accident remain at the traffic scene as potential distractors. As we have seen in the laboratory, information from the signs most often used in these situations takes longer to be integrated (Roca et al., 2012).

## General Discussion

One way to reduce the probability of accidents is by facilitating the interpretation of traffic signs. As we have seen, there are three ways to signal a T-junction: with a mandatory sign, with a prohibitory sign or with both (redundant condition). The result in all three cases is the same: one way is allowed and the other not. However, they are not cognitively equivalent. Previous studies (Castro et al., 2008; Vargas et al., 2011; Roca et al., 2012) in the laboratory have shown that inferences about allowed and not-allowed are faster when mandatory signs rather than prohibitory signs are used, other than in exceptional situations. Study 1 in this work showed that presenting the two signs, giving redundant information, was no better than presenting just the mandatory sign, although in some situations, when a prohibitory sign was used, adding a redundant mandatory sign could be useful. That is, a faster time was obtained in some conditions for the two signs in comparison with just the prohibitory sign and in no condition was the time longer for the combined signs. This result could contribute to restricting the context of usefulness of giving redundant information. The use of reiterative information could produce some negative effects regarding compliance with Variable Message Signs (Thomas and Charlton, 2020). In the context of evacuation signalling, Kwee-Meier et al. (2019) showed that the use of prohibition added to the allowed direction created confusion and should be avoided.

In Study 2, the results suggest low levels of agreement among those responsible for signing. Actually, some of them said they did not have a theoretical or empirical base from which to follow a clear criterion. Despite the apparent lack of agreement, the average frequency is higher for prohibitory signs and in a few cases for double signing. The differences were even greater for four-way intersections. It is important to note that these results contrast with those obtained in the laboratory, which showed faster processing when inferences were based on mandatory signs.

There are some limitations in the present work. We asked those responsible for signalling in the main cities of Spain to participate, and we think that the number was high enough to obtain a good picture, although a greater number of participants would have been desirable. Another obvious limitation is that there are many factors (external and internal) when driving that could influence the preference for using one way of signalling or the other. From the experimental research reviewed for this paper, we have mentioned some important factors that have been studied but there are other potential factors in real settings. Future research could provide new and convergent evidence about drivers’ inferences with traffic signs. For example, using driving simulators would help test ecological conditions. In particular, we could test a prediction derived from our view: no differences are shown, which depends on presenting our scenes in egocentric rather than allocentric view. Our approach was based on predictions from deductive theories. These theories postulate a conversion from diagrammatic premises to symbolic representations before other inference processes run. Depending on the theory, these could be mental models, propositions or probabilities. Since the same symbolic representations are expected from allocentric and egocentric presentations (right turn not-allowed…), no differences are predicted in the inference. Also, by analysing participants’ eye movements, different strategies for processing could be tested. Thus, it is possible that these measures would allow capture of the processing of the denied location such as prohibitory right (right is not-allowed), which leads to looking to the left. Finally, a mental load framework could also be added to represent better the real traffic conditions. In any case, our approach was to analyse the basic inferences involved in the processing of the two signs in the experimental conditions described.

In conclusion, our results point to some recommendations to potentially facilitate and speed up drivers’ interpretation of traffic signs and the inferences they make from them, which would potentially give them some extra time that could be crucial to process other important information and reduce the probability of accidents. In particular, according to our results, whenever possible, we should use mandatory signs at T-junctions and, if prohibitory signs are used, we should add mandatory signs, even though they may be redundant.

## Data Availability Statement

The original contributions presented in the study are included in the article/supplementary material, further inquiries can be directed to the corresponding author.

## Ethics Statement

This study was approved by the University Ethics Committee (Comité de Ética en Investigación Humana de la Universidad de Granada: 1068/CEIH/2020). In addition, the participants provided their written informed consent to participate in this study.

## Author Contributions

CV: conceptualisation, methodology, software, investigation, writing-original preparation, and writing – review and editing. SM-R: funding acquisition, conceptualisation, methodology, software, investigation, writing-original preparation, and writing – review and editing. All authors contributed to the article and approved the submitted version.

## Funding

This work was supported by the Spanish Government, Ministry of Economy and Competitiveness (PGC2018-095868-B-I00).

## Conflict of Interest

The authors declare that the research was conducted in the absence of any commercial or financial relationships that could be construed as a potential conflict of interest.

## Publisher’s Note

All claims expressed in this article are solely those of the authors and do not necessarily represent those of their affiliated organizations, or those of the publisher, the editors and the reviewers. Any product that may be evaluated in this article, or claim that may be made by its manufacturer, is not guaranteed or endorsed by the publisher.
